# The Impact of Carotid Endarterectomy on Choriocapillaris Perfusion

**DOI:** 10.1167/iovs.64.15.42

**Published:** 2023-12-28

**Authors:** Sandy W. Zhou, Yi Zhang, Natalie Noam, David Rabinovitch, Davidov Bar, Basheer S. Yousif, Robert O'Brien, Farhan E. Hiya, Yufen Lin, Alessandro Berni, Giovanni Gregori, Ruikang K. Wang, Philip J. Rosenfeld, Omer Trivizki

**Affiliations:** 1 Department of Ophthalmology, Bascom Palmer Eye Institute, University of Miami Miller School of Medicine, Miami, Florida, United States; 2 Department of Ophthalmology, Tan Tock Seng Hospital, National Health Group Eye Institute, Singapore; 3 Department of Bioengineering, University of Washington, Seattle, Washington, United States; 4 Department of Vascular Surgery, Tel Aviv Medical Center, University of Tel Aviv, Tel Aviv, Israel; 5 Department of Ophthalmology, Tel Aviv Medical Center, University of Tel Aviv, Tel Aviv, Israel; 6 Department of Ophthalmology, University of Washington, Seattle, Washington, United States

**Keywords:** carotid endarterectomy, choriocapillaris perfusion, SS-OCTA

## Abstract

**Purpose:**

The impact of carotid endarterectomy (CEA) on choriocapillaris (CC) perfusion was investigated using swept-source optical coherence tomography angiography (SS-OCTA) imaging before and after surgery in patients with clinically significant carotid artery stenosis (CAS).

**Methods:**

In this prospective observational study, patients with clinically significant CAS undergoing unilateral CEA had SS-OCTA imaging performed in both eyes before and within 1 week after surgery. The percent CC flow deficits (CC FD%) and CC thickness were assessed using previously validated algorithms. Multivariable regression analysis was conducted to evaluate the impact of variables on the change in CC measurements.

**Results:**

A total of 112 eyes from 56 patients with an average age of 72.6 ± 6.9 years were enrolled. At baseline, significantly higher CC FD% and thinner CC thickness were observed on the surgical side (eyes ipsilateral to the side of CEA) versus the nonsurgical side (eyes contralateral to the side of CEA) (*P* = 0.001 and *P* = 0.03, respectively). Following CEA, a significant reduction in CC FD% and a significant increase in CC thickness were detected on the surgical as compared with the nonsurgical side (*P* = 0.008 and *P* = 0.01, respectively). Smoking status positively affected CC FD% change (coefficient of variation [CV] = 0.84, *P* = 0.01) on the surgical side and negatively affected CC thickness change on both the surgical side (CV = −0.382, *P* = 0.009) and the nonsurgical side (CV = −0.321, *P* = 0.04). The degree of stenosis demonstrated a positive influence on CC FD% change (CV = 0.040, *P* = 0.02) on the surgical side.

**Conclusions:**

Unilateral CEA on the side of clinically significant CAS increases carotid blood flow, which further results in improved CC perfusion.

Stenosis of the carotid artery due to atherosclerosis accounts for 10% to 20% of all strokes.^1^ Clinically significant carotid artery stenosis (CAS) is typically defined as a narrowing of the carotid artery over 50% to 60%.^2^^,^^3^ Because the flow within the ophthalmic artery (OA), the first intradural branch of the carotid artery, depends on carotid artery blood flow, clinically significant CAS can result in impaired flow dynamics of ocular circulation.^4^^–^^7^ This impaired flow may lead to ocular symptoms and signs on the ipsilateral side of the affected carotid artery, including amaurosis fugax or ocular ischemic syndrome (OIS).^6^^,^^7^ These ocular manifestations of impaired OA blood flow can be the initial warning signs of a potential cerebral infarction with devastating consequences.^7^ In cases of severe CAS, several studies using Doppler ultrasound have shown a decreased or absent blood flow in the OA.^4^ Additionally, a study using four-dimensional (4D) flow magnetic resonance imaging (MRI) of the OA revealed retrograde flow in half of patients with severe CAS.^5^

Carotid endarterectomy (CEA) is a highly effective treatment for directly removing plaque from the carotid in patients with moderate to severe CAS.^8^ Recent studies have shown that CEA can be associated with significant blood flow restoration in the OA, correction of reversed OA flow, and clinical improvement in ischemic ophthalmic symptoms in patients with OIS.^4^^,^^6^^,^^9^^,^^10^ However, the methods used for measuring changes in ocular blood flow before and after CEA, such as Doppler ultrasound or 4D flow MRI, are not readily available in ophthalmology clinics. Furthermore, there are concerns about the reliability and reproducibility of these sophisticated tests, as the devices and technical expertise required to perform these tests are not readily available. Therefore, alternative strategies are being explored that can be more easily performed in ophthalmology clinics and serve as a direct measure of ocular circulation. Such strategies should be useful for studying changes in the downstream ocular perfusion in patients with CAS to help determine when CEA is needed and to document any beneficial changes in OA blood flow after CEA.

Given that the choroid is primarily supplied by the posterior ciliary arteries and these branches off the OA are responsible for roughly 85% of all ocular blood flow,^11^ there has been growing interest in studying changes in choroidal perfusion following CEA in patients with clinically significant CAS. Choroidal thickness (CT), which depends upon the choroidal vascular volume, is considered to be a surrogate for choroidal perfusion.^12^ Spectral-domain optical coherence tomography (SD-OCT) imaging using the enhanced depth imaging strategy or swept-source OCT (SS-OCT) imaging using an optical attenuation correction strategy have been used successfully to measure CT.^12^^,^^13^ CT has been assessed in several studies involving patients with CAS both before and after CEA using either single B-scans or volume raster scans.^14^^–^^20^ So far, the results have been inconclusive.

In our previous study, we investigated changes in CT after CEA using SS-OCT imaging and found a significant increase in CT in eyes on the same side as CEA, whereas no changes were detected in the contralateral eye.^21^ Given these results, we proposed that the changes in choriocapillaris (CC) perfusion might serve as another vascular parameter that could demonstrate improvements in ocular blood flow after CEA.

The CC is the innermost layer of the choroid, responsible for direct nourishment of the retinal pigment epithelium (RPE) and photoreceptors.^22^^,^^23^ Although the CC represents a relatively small portion of the choroid in terms of vascular volume, it serves as the only capillary bed in the choroid and has been shown to have an important role in the pathogenesis of age-related macular degeneration (AMD), polypoidal choroidal vasculopathy (PCV), central serous chorioretinopathy (CSCR), diabetic retinopathy, and myopia.^22^ Because the CC represents the terminal capillaries of the choroid and connects the choroidal arterial and venous circulations,^24^^,^^25^ we hypothesized that the blood flow within the CC might be a sensitive measure of alterations in blood flow into the choroid. Moreover, studying the change in CC blood flow following CEA may provide insights into the potential therapeutic ocular benefits from CEA when treating patients with significant CAS.

To investigate CC perfusion in patients before and after CEA, we used SS-OCT angiography (SS-OCTA) imaging to quantify the percent CC flow deficits (CC FD%)^26^^–^^28^ and CC thickness.^29^ In this study, we report a decrease in CC FD% and an increase in CC thickness within 1 week of CEA.

## Methods

This prospective OCTA imaging study was approved by the ethics committee of the Tel Aviv Medical Center, University of Tel Aviv, in Israel. Informed consent was obtained from each subject. This study was performed in accordance with the tenets of the Declaration of Helsinki and complied with the Health Insurance Portability and Accountability Act of 1996.

Patients diagnosed with clinically significant CAS undergoing unilateral CEA were enrolled in the study between January 2021 and December 2022. Diagnosis of CAS was made using ultrasound, and the North American Symptomatic Carotid Endarterectomy Trial (NASCET) stenosis classification was used for stenosis grading of the internal carotid artery (ICA). Low-degree stenosis is 0% to 40%, moderate stenosis is 50% to 60%, and severe stenosis is more than 70%, which is also hemodynamically relevant stenosis. Clinically significant CAS is defined as more than 50% stenosis.^30^

Each patient's demographics, including gender, age, extent of stenosis of both sides, comorbidities including diabetes and hypertension, status of smoking, and ophthalmic examination, including best-corrected visual acuity (BCVA; Early Treatment Diabetic Retinopathy Study [ETDRS] letter score) and low-luminance BCVA, were recorded. Systolic blood pressure (SBP) and diastolic blood pressure (DBP) measurements at both baseline and after CEA were also documented. Mean arterial pressure (MAP) was calculated as DBP + 1/3(SBP – DBP). Patients were excluded from this study if they were unable to tolerate ocular imaging, if the presence of ocular media severely affected the ability of OCT to visualize the retina in either eye resulting in poor image quality (e.g., significant central corneal scarring, lens opacities along visual axis, posterior capsule opacification), or if there were conditions that could affect CC assessment (e.g., retinal vascular occlusions; exudative AMD; PCV or geographic atrophy; CSCR; choroiditis; vitreomacular changes such as traction or an epiretinal membrane; a history of retinal detachment, serious ocular trauma, previous retinal laser therapy, any vitreoretinal surgery, high refractive error, or glaucoma).

Enrolled patients had both eyes scanned using a SS-OCTA instrument (PLEX Elite 9000; Carl Zeiss Meditec, Dublin, CA, USA) at baseline 1 to 2 days before CEA surgery and at postoperative follow-up which was within 1 week after CEA surgery. In order to minimize the influence of diurnal variations, we conducted all scans during the same morning clinic session. Images were acquired using the OCTA 6 × 6-mm scanning protocol centered on the fovea. Scans with a signal strength below 7 were excluded because this range was considered the lower boundary of signal strength for reliable imaging, especially in clinical studies and is a cut-off recommended by manufacturers. All OCT scans were normalized against a signal strength score of 9 to minimize the signal strength variation between scans.^31^ The SS-OCTA instrument has a central wavelength of 1050 nm, a bandwidth of 100 nm, and a scanning rate of 100 kHz, providing an axial resolution of ∼5 µm in tissue and a lateral resolution at the retinal surface estimated at approximately 20 µm. The scans consist of 500 A-scans per B-scans with two repeated B-scans at the same B-scan position to generate the OCTA images using the published complex optical microangiography (OMAG) algorithm.^32^ For each A-scan, it was sampled with 1024 pixels, meaning each pixel in *z*-dimension was ∼2 µm in tissue. The study eye was chosen based on the side where the CEA was performed. The side ipsilateral to the CEA is referred to as the surgical side, and the side contralateral to the CEA is referred to as the nonsurgical side.

The OCTA scans were used to compute the following parameters based on our previously validated semi-automated algorithms: (1) CC FD%, defined as the percentage of pixels representing flow deficits relative to all the pixels within a given region^26^^–^^28^^,^^33^^–^^35^; (2) CC thickness^29^; and (3) mean choroidal thickness (MCT).^12^

Following the published guidelines for the assessment of CC flow deficits,^36^ the CC en face flow images were generated using a 16-µm-thick slab with its inner and outer boundaries located at 4 µm and 20 µm beneath Bruch's membrane (BM), respectively. BM was first identified using an automated algorithm with some manual adjustment when necessary. To account for signal loss in the CC en face flow images due to the overlying anatomy, a compensation strategy using the corresponding CC en face structural images was adopted, and retinal vessel projection artifacts were removed for more accurate quantification.^27^ The fuzzy C-means method was then applied to the CC en face flow images with normalization to generate the CC FD binary maps using adaptive thresholds and flow deficits with a diameter smaller than 24 µm were removed from the maps to reduce noise.^33^ CC FD% was analyzed in circles centered on the fovea with diameters of 3 mm and 5 mm within the 6 × 6 scan pattern. The fovea center was identified by the algorithm and manually confirmed on the OCTA en face images.

For the CC thickness analysis, structural information was used to flatten the OCTA scans at the RPE centerline.^37^^,^^38^ Then A-scan signals were normalized by local averaging, and the position of the OCTA CC flow peak along each A-scan was determined. Mean CC thickness was measured as the full width at half maximum of this peak.^29^

MCT was measured using our previously validated automated method. Briefly, attenuation correction was applied to the structural component of the OCTA scans to enhance the contrast at the choroidal–sclera interface.^39^ The boundaries of both BM and the choroidal–scleral interface were segmented on the optical attenuation coefficient–corrected OCT dataset. The distance between the segmented BM and the boundary of the choroidal-scleral interface was then averaged to calculate the MCT for each volume scan.^37^

Statistical analysis was performed using SAS 9.4 (SAS Institute, Cary, NC, USA) and R 4.2.2 (R Foundation for Statistical Computing).^40^ Normality was assessed using normal quantile–quantile plots. Results are expressed as mean ± standard deviation (SD), if applicable. The statistical significance of differences between the two sides (surgical vs. nonsurgical) was assessed with the clustered Wilcoxon rank-sum test using the Rosner–Glynn–Lee method.^41^^,^^42^ The statistical significance of differences between before and after CEA on one side (within group) was assessed with the Wilcoxon signed-rank test. Multivariable regression analysis was used to assess the impact of variables including age, MAP, degree of stenosis, diabetes, hypertension, and status of smoking on the changes of CC FD% and CC thickness. *P* < 0.05 was considered statistically significant.

## Results

A total of 112 eyes of 56 patients (80% male) with clinically significant CAS undergoing unilateral CEA were enrolled in the study with a mean age of 72.6 ± 6.9 years. Among the patients, 50% had diabetes, 77% had hypertension, and more than half of the patients (57%) were smokers. The MAP was 91.95 ± 11.10 mmHg at baseline, compared to 86.94 ± 11.99 mmHg after CEA (*P* = 0.002). The mean extent of carotid artery stenosis was 85.47% ± 9.90% on the surgical side compared to 36.71% ± 22.94% on the nonsurgical side (*P* < 0.001). The BCVA at baseline was 45.65 ± 5.51 ETDRS letters on the surgical side versus 47.80 ± 6.65 ETDRS letters on the nonsurgical side (*P* = 0.09). The low-luminance BCVA was 24.44 ± 6.66 ETDRS letters on the surgical side versus 28.71 ± 11.12 ETDRS letters on the nonsurgical side (*P* = 0.10). The average follow-up time (time from surgery to postoperative follow up) was 2.71 ± 1.36 days. The demographic features and other characteristics of the patients are provided in Table 1.

**Table 1.** Baseline Features of Participants

General Characteristics			
Age (y), mean ± SD	72.63 ± 6.85		
Male gender, *n* (%)	45 (80)		
Diabetes, *n* (%)	28 (50)		
Hypertension, *n* (%)	43 (77)		
Smoking, *n* (%)	32 (57)		
	Surgical	Nonsurgical	*P*
Stenosis degree (%), mean ± SD	85.47 ± 9.90	36.71 ± 22.94	<0.001^*^
Ophthalmic parameters, mean ± SD			
BCVA	45.65 ± 5.51	47.8 ± 6.65	0.09
Low-luminance BCVA	24.44 ± 6.66	28.71 ± 11.12	0.10
OCTA parameters, mean ± SD			
CC FD% in 3-mm circle	13.05 ± 3.81	12.03 ± 4.22	0.01^*^
CC FD% in 5-mm circle	10.93 ± 2.26	10.17 ± 2.51	0.001^*^
CC thickness (µm) in 3-mm circle	6.53 ± 1.08	6.64 ± 0.91	0.08
CC thickness (µm) in 5-mm circle	6.78 ± 1.02	6.93 ± 0.88	0.03^*^

*P < 0.05.

At baseline, a significantly higher CC FD% was seen on the surgical side compared with the nonsurgical side in both the 3-mm circle (13.05% ± 3.81% vs. 12.03% ± 4.22%; *P* = 0.01) and the 5-mm circle (10.93% ± 2.26% vs. 10.17% ± 2.51%; *P* = 0.001) centered on the fovea. Although the CC thickness was thinner on the surgical side than the nonsurgical side in both the 3-mm circle (6.53 ± 1.08 µm vs. 6.64 ± 0.91 µm; *P* = 0.08) and the 5-mm circle (6.78 ± 1.02 µm vs. 6.93 ± 0.88 µm; *P* = 0.03), a statistically significant difference between the two sides was only seen in the 5-mm circle. Baseline CC FD% and CC thickness are summarized in Table 1.

After CEA, the CC FD% results within the 3-mm circle centered on the fovea did not show a significant reduction on the surgical side (12.74% ± 3.38% after CEA, corresponding to an average reduction of 0.31% ± 1.49%) as compared with the nonsurgical side (12.06% ± 4.03% after CEA, corresponding to an average increase of 0.03% ± 2.29%; *P* = 0.11). However, in the 5-mm circle centered on the fovea, there was a significant reduction in the CC FD% on the surgical side (10.57% ± 2.07% after CEA, corresponding to an average reduction of 0.36% ± 1.17%) as compared with the nonsurgical side (10.20% ± 2.21% after CEA, corresponding to an average increase of 0.03% ± 1.92%; *P* = 0.008).

Following CEA, the 3-mm circle showed a statistically significant increase in the mean CC thickness on the surgical side (6.81 ± 1.17 µm after CEA, corresponding to an average increase of 0.28 ± 0.59 µm) as compared with the nonsurgical side (6.73 ± 1.02 µm after CEA, corresponding to an average increase of 0.09 ± 0.60 µm; *P* = 0.04). This significant increase in CC thickness was also seen in the 5-mm circle (7.05 ± 1.12 µm after CEA, corresponding to an average increase of 0.27 ± 0.54 µm) on the surgical side as compared with the nonsurgical side (7.00 ± 1.00 µm after CEA, corresponding to an average increase of 0.06 ± 0.53 µm; *P* = 0.01).

**Figure 1.**
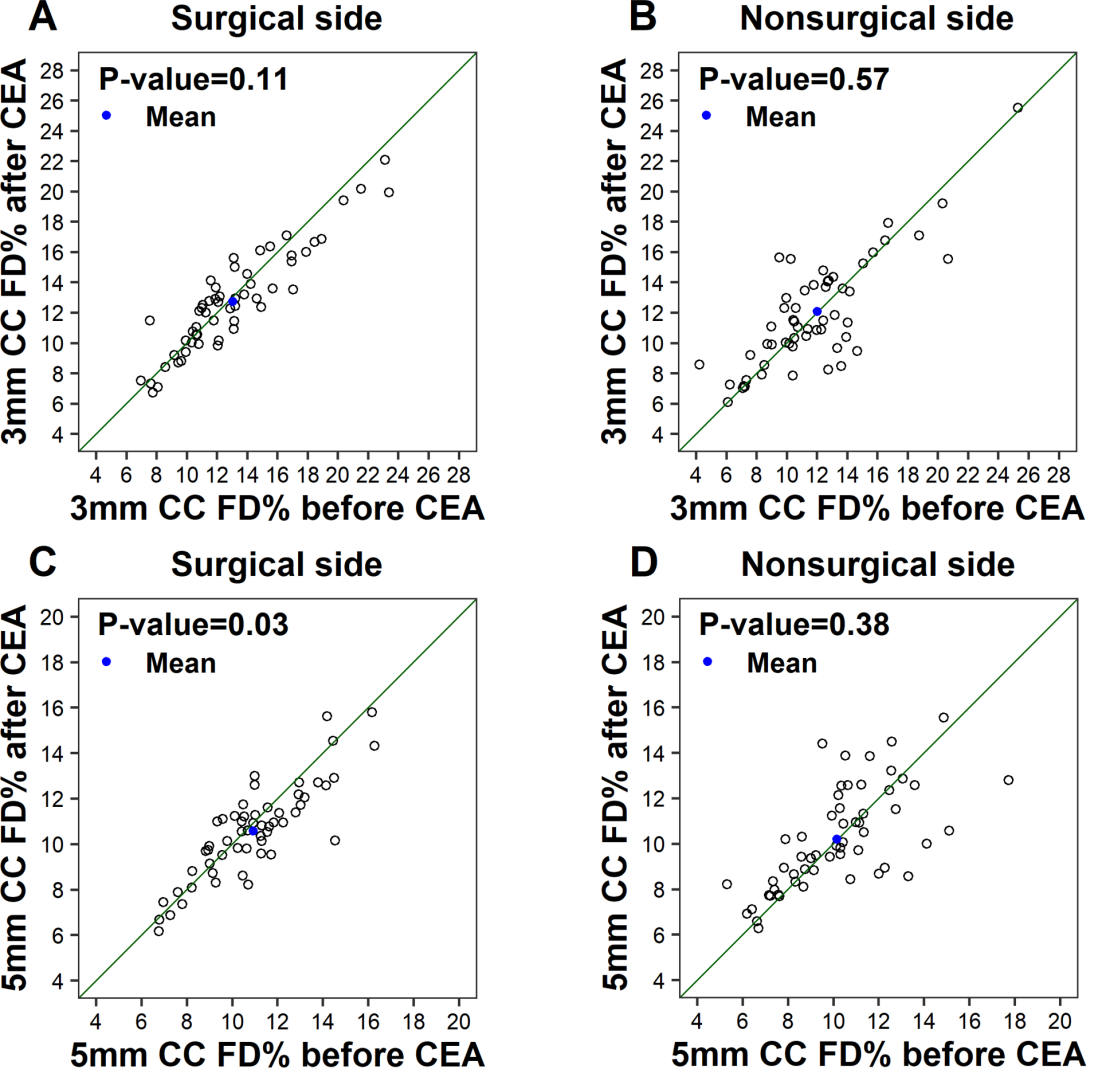
Scatterplots of CC FD% within the 3-mm circle and 5-mm circle centered on the fovea before versus after CEA. The *blue dots* indicate the mean CC FD% values before and after CEA. The *diagonal line* is a 1:1 reference line.

**Figure 2.**
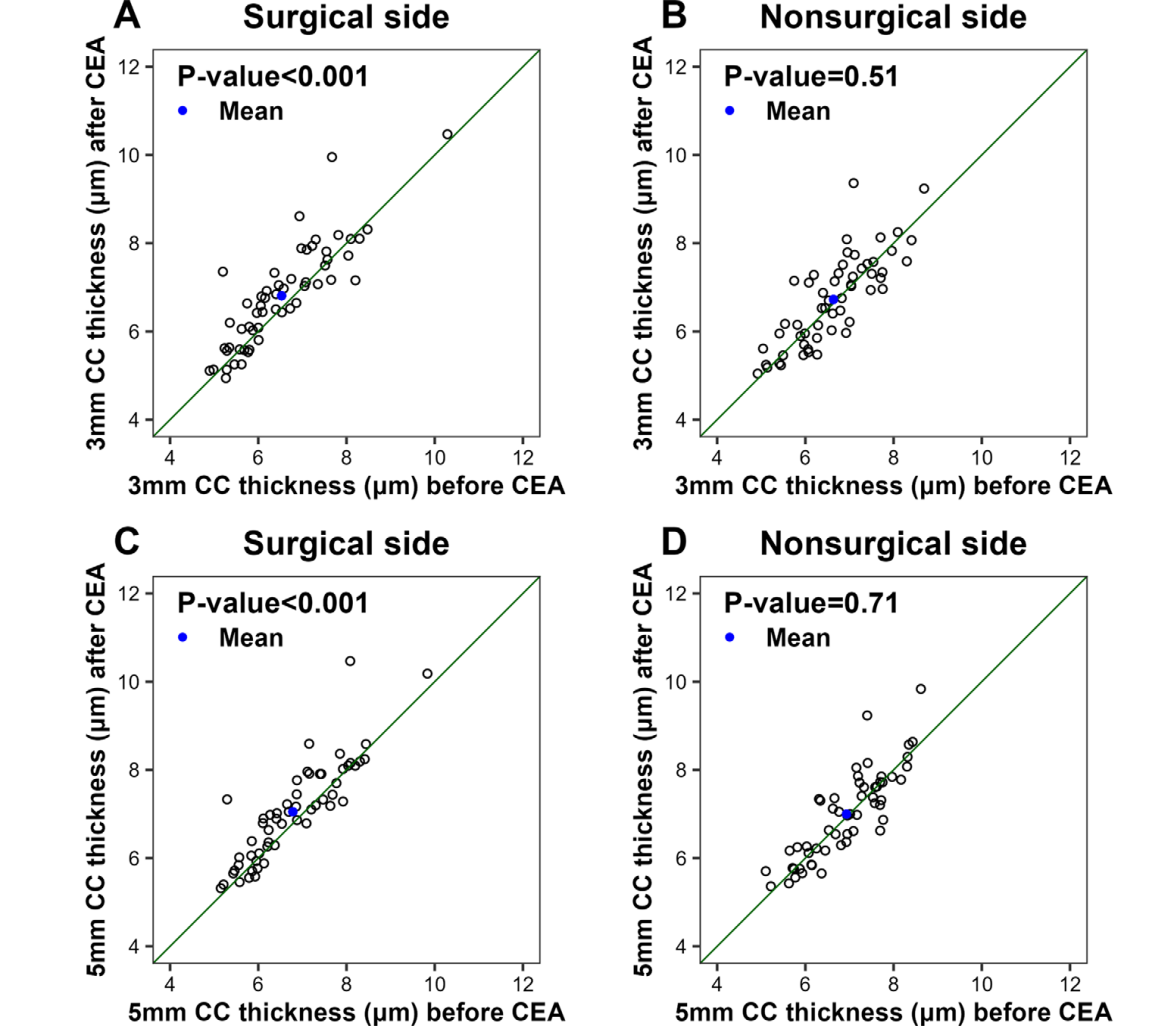
Scatterplots of CC thickness (µm) within the 3-mm circle and 5-mm circle centered on the fovea before versus after CEA. The *blue dots* indicate the mean CC thickness values before and after CEA. The *diagonal line* is a 1:1 reference line.

An analysis of the changes in CC FD% and CC thickness before and after CEA on each side (Table 2) showed that the CC FD% results within the 3-mm circle centered on the fovea did not show significant differences on either the surgical side (*P* = 0.11) or the nonsurgical side (*P* = 0.57) (Figs. 1A, 1B). However, in the 5-mm circle centered on the fovea, the CC FD% on the surgical side after CEA was significantly lower than before CEA (*P* = 0.03) (Fig. 1C). In contrast, in the 5-mm circle on the nonsurgical side, the CC FD% after CEA was not statistically different from CC FD% before CEA (*P* = 0.38) (Fig. 1D). Moreover, the mean CC thickness on the surgical side was significantly greater following CEA than it was before CEA, as observed in both the 3-mm circle (*P* < 0.001) and the 5-mm circle (*P* < 0.001) (Figs. 2A, 2C). No statistically significant differences between CC thickness after CEA versus before CEA were observed on the nonsurgical side in either the 3-mm circle (*P* = 0.51) or the 5-mm circle (*P* = 0.71) (Figs. 2B, 2D). In addition, we found a significant correlation between the changes of CC FD% and the changes of CC thickness on both surgical (*r* = −0.28, *P* = 0.03) and nonsurgical sides (*r* = −0.27, *P* = 0.04) in the 5-mm circle.

**Table 2.** CC FD% and CC Thickness Before and After CEA

Parameters	Before CEA	After CEA	Change	*P*
Surgical side, mean ± SD				
CC FD% in 3-mm circle	13.05 ± 3.81	12.74 ± 3.38	−0.31 ± 1.49	0.11
CC FD% in 5-mm circle	10.93 ± 2.26	10.57 ± 2.07	−0.36 ± 1.17	0.03^*^
CC thickness (µm) in 3-mm circle	6.53 ± 1.08	6.81 ± 1.17	0.28 ± 0.59	<0.001^*^
CC thickness (µm) in 5-mm circle	6.78 ± 1.02	7.05 ± 1.12	0.27 ± 0.54	<0.001^*^
Nonsurgical side, mean ± SD				
CC FD% in 3-mm circle	12.03 ± 4.22	12.06 ± 4.03	0.03 ± 2.29	0.57
CC FD% in 5-mm circle	10.17 ± 2.51	10.20 ± 2.21	0.03 ± 1.92	0.38
CC thickness (µm) in 3-mm circle	6.64 ± 0.91	6.73 ± 1.02	0.09 ± 0.60	0.51
CC thickness (µm) in 5-mm circle	6.93 ± 0.88	7.00 ± 1.00	0.06 ± 0.53	0.71

* *P* < 0.05.

**Figure 3.**
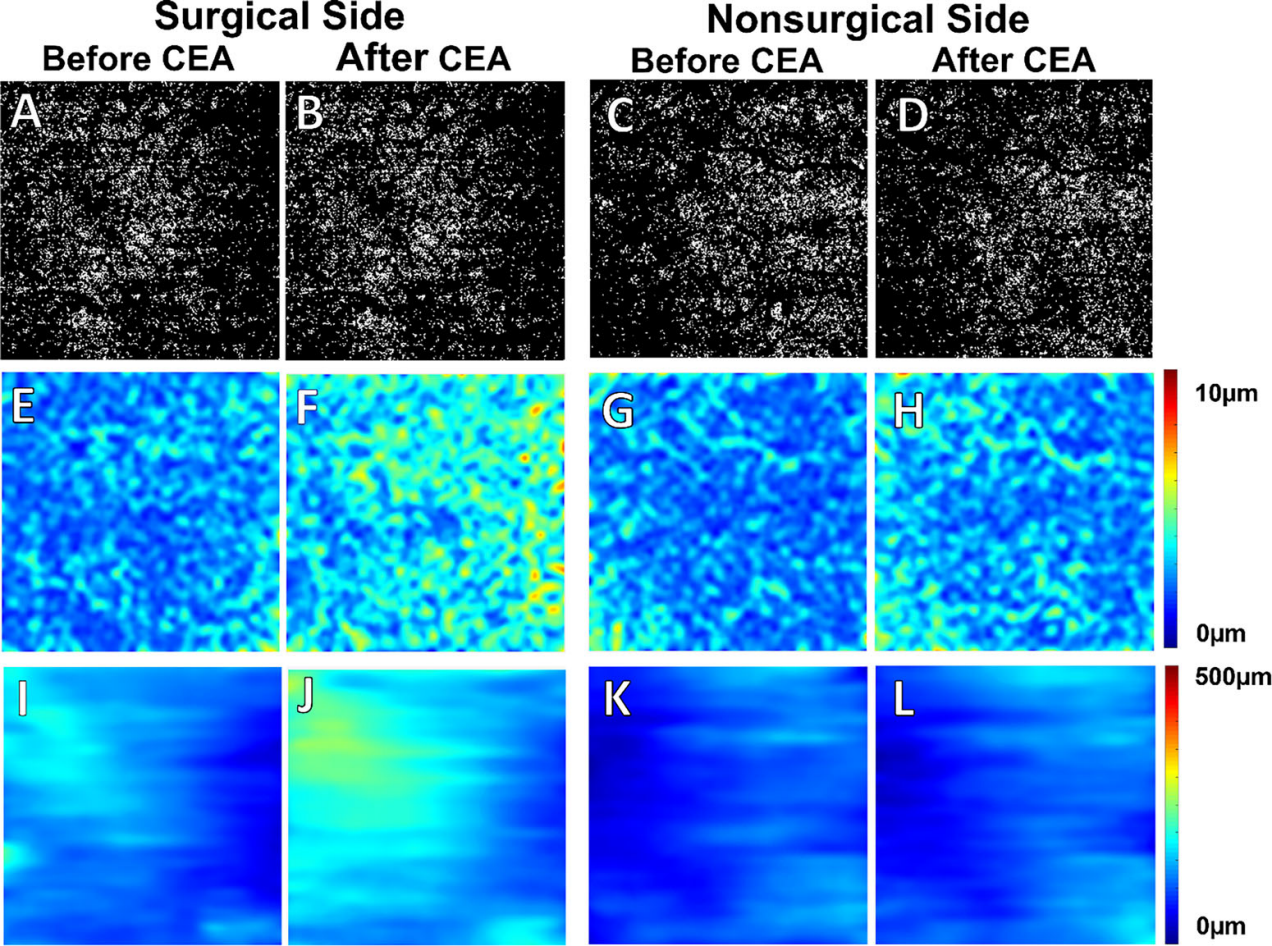
Case 1. An 80-year-old subject with 95% CAS on the surgical side and 20% CAS on the nonsurgical side underwent unilateral CEA on the ipsilateral surgical side. (**A**–**D**) CC FD% maps. (**E**–**H**) CC thickness maps. (**I**–**L**) CT maps. On the surgical side, we did not observe visually significant change of the CC FD% in the binary maps before or after CEA despite a reduction in the CC FD% measurements (14.15% before CEA vs. 12.58% after CEA; **A** vs. **B**). Significantly thin CC thickness maps (**E**, **G**) and CT maps (**I**, **K**) were seen at baseline on both sides. Following CEA, there was an apparent increase in both CC thickness (5.29 µm before CEA vs. 7.33 µm after CEA; **E** vs. **F**) and CT (117.7 µm before CEA vs. 161.81 µm after CEA; **I** vs. **J**) maps on the surgical side. On the nonsurgical side, there was no visible change noted in the CC FD% binary maps before and after CEA (13.61% before CEA vs. 12.58% after CEA; **C** vs. **D**), but there was a slight increase in CC thickness after CEA (5.10 µm before CEA vs. 5.70 µm after CEA; **G** vs. **H**). No obvious change was observed in the CT maps before and after CEA on the nonsurgical side (92.80 µm before CEA vs. 99.76 µm after CEA; **K** vs. **L**).

**Figure 4.**
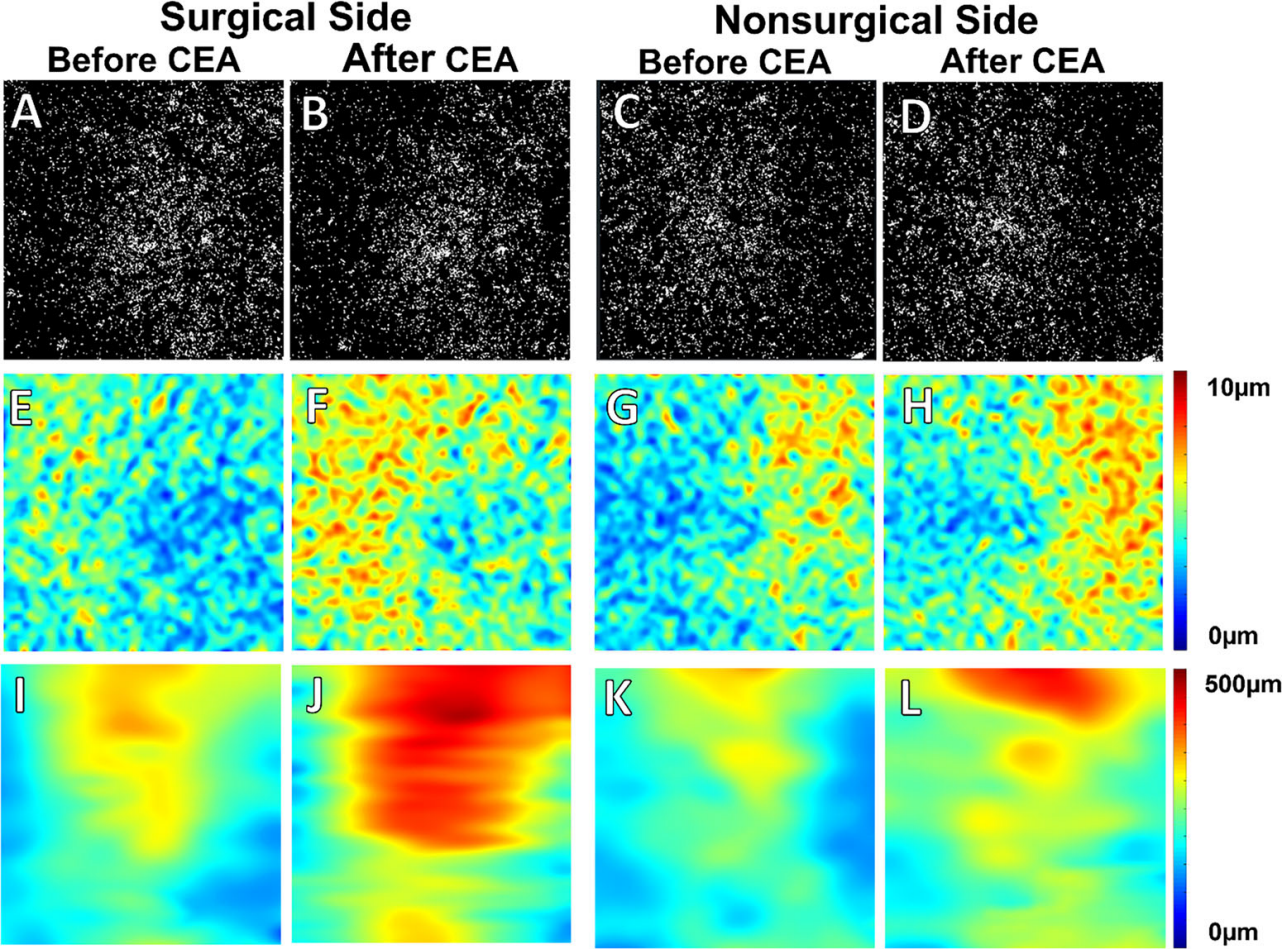
Case 2. A 67-year-old subject with 85% CAS on the surgical side and 50% CAS on the nonsurgical side underwent unilateral CEA on the ipsilateral surgical side. (**A**–**D**) CC FD% maps. (**E**–**H**) CC thickness maps. (**I**–**L**) CT maps. On the surgical side, no visually significant difference was seen between CC FD% maps before and after CEA, although the CC FD% measurement had a minor decrease after CEA: 12.09% (**A**) versus 11.35% (**B**). After the CEA, there was a significant increase in the CC thickness (8.08 µm before CEA vs. 10.47 µm after CEA; **E** vs. **F**) and in the CT (250.17 µm before CEA vs. 366.11 µm after CEA; **I** vs. **J**) maps. On the nonsurgical side, there was no observable change in the CC FD% maps before and after CEA (11.15% before CEA vs. 10.92% after CEA; **C** vs. **D**). The CC thickness map showed a less prominent but noticeable increase after CEA (8.62 µm before CEA vs. 9.84 µm after CEA; **G** vs. **H**), with a similarly observable increase in the CT map superiorly (227.11 µm before CEA vs. 296.22 µm after CEA; **K** vs. **L**).

Figures 3 and 4 depict two noteworthy instances of a visually minor reduction in the CC FD% binary maps on the surgical side following CEA. Concurrently, there were prominent increases in CC thickness maps on the surgical side, coinciding with significant increases in choroidal thickness maps. In addition to the changes on the surgical side, the second case (Fig. 4) also exhibited a simultaneous increase in both CC thickness and choroidal thickness on the nonsurgical side, although to a lesser extent than on the surgical side.

Multivariable regression analyses were performed to assess the effects of different baseline variables, including age, MAP, degree of stenosis, presence of diabetes, hypertension, and smoking status on changes for both the CC FD% and CC thickness measurements before and after CEA in the 5-mm circle. These analyses showed that age, MAP, and the presence of comorbidities such as diabetes and hypertension had no discernible impact on the changes in CC FD% and CC thickness before and after CEA. However, smoking status was found to have a positive effect on CC FD% changes after CEA (coefficient of variation [CV] = 0.84, *P* = 0.01) on the surgical side and a negative effect on CC thickness changes on both the surgical side (CV = −0.382, *P* = 0.009) and the nonsurgical side (CV = −0.321, *P* = 0.04) after CEA. In addition, the extent of stenosis exhibited a positive influence on the changes of CC FD% (CV = 0.040, *P* = 0.02) after CEA on the surgical side, although no significant association was observed between the extent of stenosis and changes in CC thickness (Tables 3, 4).

**Table 3.** Multivariable Analysis of the Role of Baseline Variables on Change of CC FD% in 5-mm Circle Before and After CEA

	Surgical Side		Nonsurgical Side	
Baseline Variables	Coefficient (95% CI)	*P*	Coefficient (95% CI)	*P*
Age	−0.001 (−0.048, 0.045)	0.96	0.007 (−0.076, 0.091)	0.86
MAP	0.001 (−0.028, 0.030)	0.94	−0.018 (−0.070, 0.033)	0.47
Extent of stenosis	0.040 (0.008, 0.072)	0.02	−0.006 (−0.032, 0.019)	0.62
Diabetes	−0.025 (−0.698, 0.648)	0.94	−0.804 (−1.977, 0.370)	0.17
Hypertension	−0.104 (−0.901, 0.694)	0.80	0.378 (−1.013, 1.768)	0.59
Smoking	0.84 (0.184, 1.503)	0.01	1.056 (−0.079, 2.190)	0.07

CI, confidence interval.

**Table 4.** Multivariable Analysis of the Role of Baseline Variables on Change of CC Thickness (µm) in 5-mm Circle Before and After CEA

	Surgical Side		Nonsurgical Side	
Baseline Variables	Coefficient (95% CI)	*P*	Coefficient (95% CI)	*P*
Age	0.006 (−0.015, 0.026)	0.58	−0.013 (−0.036, 0.009)	0.25
MAP	0.004 (−0.008, 0.017)	0.50	0.003(−0.011, 0.016)	0.69
Extent of stenosis	−0.003 (−0.017, 0.011)	0.66	−0.001 (−0.008, 0.005)	0.90
Diabetes	−0.208 (−0.500, 0.085)	0.16	0.019 (−0.296, 0.334)	0.90
Hypertension	0.304 (−0.042, 0.650)	0.08	0.083 (−0.291, 0.456)	0.66
Smoke	−0.382 (−0.668, −0.096)	0.009	−0.321 (−0.626, −0.016)	0.04

## Discussion

 In this report, we explored the changes in CC measurements in eyes on both the surgical side and the nonsurgical side following unilateral CEA. At baseline, significantly higher CC FD% was seen with significantly thinner CC thickness on the surgical side, indicating a decline in CC perfusion on the side of stenosis. After CEA, a significant decrease in CC FD% was accompanied by a significant increase in CC thickness on the surgical side, indicating an immediate improvement in ocular perfusion with increased carotid artery flow.

These baseline results indicate that there could be a sizeable and sustained decline in CC perfusion as evidenced by higher CC FD% and thinner CC thickness on the side of the stenosis, even in the absence of symptoms or clinical indicators of OIS such as amaurosis fugax or retinal hemorrhages. To date, only two other studies have investigated CC perfusion in patients undergoing CAS using different parameters. Wan et al.^14^ assessed the mean perfusion of the CC, which was defined as the length of microvessels of the perfused choriocapillaris per unit area in square millimeters (mm^2^). They did not observe a statistically significant difference in the mean perfusion of the CC between CAS patients and healthy controls. Meanwhile, Pierro et al.^43^ analyzed the vessel density of the CC using an in-house algorithm, which also failed to reveal any differences between the ipsilateral and contralateral sides of CEA. However, these strategies are likely problematic because current OCT instruments lack the necessary imaging resolution to accurately measure microvessel length or actual vessel density in the CC. Therefore, our results cannot be directly compared with the two studies mentioned above. In our study, it is noteworthy that at baseline CC FD% was significantly higher on the surgical side versus the nonsurgical side in both the 3-mm circle and the 5-mm circle, but CC thickness was only significantly lower in the 5-mm circle and not the 3-mm circle compared to the nonsurgical side. One explanation of this seemingly more significant difference in CC FD% between the two sides at baseline than CC thickness is because measuring CC FD% yields a binary outcome. Therefore, the flow remains undetectable when it falls below the threshold. In contrast, CC thickness is viewed as a more continuous parameter. Thus, we hypothesize that the CC FD% threshold was achieved on the surgical side but not on the nonsurgical side, thereby leading to a statistically significant disparity between the two sides. On the other hand, due to the small numerical values and the persistent change of CC thickness beyond the detection of CC FD% measurements, the statistically significant difference between the surgical and nonsurgical sides remains elusive, despite the diminished flow.

Furthermore, our study found a statistically significant decrease in CC FD% measurements within the 5-mm fovea-centered circle after surgery on the surgical side, but the reduction in CC FD% measurements within the 3-mm circle did not achieve statistical significance as compared with eyes on the nonsurgical side. The reduction in CC FD% measurements in the 5-mm circle suggests that there was an overall improvement in blood flow within the CC after surgery. There are several potential explanations for the more prominent reduction of CC FD% within the 5-mm circle compared with the 3-mm circle. First, we have shown in our prior study that a considerable reduction in perfusion in the 3-mm circle as opposed to the 5-mm circle is associated with aging.^44^ If this age-related decrease in perfusion is due to capillary loss rather than a decrease in capillary perfusion, an increase in blood flow within the 3-mm circle may not lead to a reduction in CC FD%.^28^^,^^39^ Another possibility is that the CC in the central macula has greater resistance due to the presence of a denser vascular network and narrower lumens suggested by previous studies,^45^^,^^46^ which could result in decreased responsiveness to perfusion improvement in the 3-mm circle. Therefore, we propose that an age-related decrease in central macular perfusion and the likely increased resistance of central CC perfusion compared with the rest of the macula might explain our ability to detect CC reperfusion in the 5-mm circle but not in the central 3-mm circle after CEA.

To explore changes in blood volume within the CC after CEA, we measured CC thickness.^29^ In contrast to the insignificant change of CC FD% in the 3-mm circle, we detected a statistically significant increase in CC thickness in the 3-mm circle on the surgical side versus the nonsurgical side after CEA. These significant changes were also seen in the 5-mm circle of the fovea. These results indicate an immediate expansion of blood volume in the CC from the reperfused ICA. However, these findings also raised a question about the observed discrepancy between CC FD% and CC thickness changes in the 3-mm circle of the fovea, with volumetric change appearing to be more pronounced than the flow-related change. We propose that this discordance is likely due to the threshold-based approach used to measure flow deficits. Flow deficits are defined as regions of decreased or absent perfusion in which the blood flow signal is below the sensitivity limit of OCTA technology and cannot be detected. To quantify CC FD%, the OCTA CC images must be binarized, which involves applying an adaptive thresholding strategy to obtain the flow deficits.^33^ As the calculation of CC FD% involves a binary thresholding operation that relies on several factors such as the scanning speed and wavelength of the instrument, the angiographic algorithm used, signal-to-noise ratio of the flow signals, and optical scattering properties of anatomic structures overlying the CC, this approach could underestimate the actual flow improvement in areas where the baseline perfusion was already low.^29^ Therefore, the more significant increase in CC thickness may reflect restoration of the blood volume in these areas, which is not necessarily proportional to the magnitude of the flow improvement. In addition, if some of the CC has been lost due to aging, then increased perfusion through the remaining capillaries would increase the thickness without changing the FD%.

However, our analysis still lacks the quantitative assessment of CC blood flow velocity, which could provide additional information about the hemodynamic changes occurring in the CC after CEA. Alterations in blood flow velocities in the retinal microvasculature have been observed in various retinal vascular conditions such as diabetic retinopathy, retinal vein occlusion, and sickle cell disease using different technologies such as variable interscan time analysis (VISTA) algorithm, laser speckle flowgraphy, and, most recently, adaptive optics scanning laser ophthalmoscopy.^47^^–^^50^ Therefore, the alteration in flow velocity within the CC may serve as another important indicator of perfusion improvement after carotid artery revascularization.

Although there are no comparable studies investigating changes in CC FD% and CC thickness measurements after CEA, our results are supported by studies demonstrating increases in CT measurements after carotid surgery.^14^^–^^18^ Most of these studies used SD-OCT imaging,^15^^–^^20^ except for one study that employed SS-OCT imaging.^14^ Overall, two studies found an increase in CT following CEA during the acute postoperative follow-up period within 1 week,^14^^,^^15^ but other research groups reported that CT increased with longer term postoperative follow-up, ranging from 1 to 6 months, but not during the acute postoperative period.^16^^–^^18^ Two other studies did not observe any change in CT before and after CEA on either the ipsilateral side of the CEA or the contralateral side.^19^^,^^20^ Ala-Kauhaluoma et al.^19^ attributed the absence of a response to the lack of vessel elasticity in severe cases, whereas Rabina et al.^20^ proposed that the lack of a choroidal response could be due to the presence of sufficient artery perfusion before surgery due to mild CAS. However, neither hypothesis was supported by flow assessments of the carotid or ophthalmic arteries. In our recently published paper using the same cohort of patients,^21^ we demonstrated significantly increased MCT on the surgical side after CEA. It is noteworthy that Pearson's correlation analyses of our series showed a moderately positive correlation between the changes in CC thickness and the changes in MCT across all cases after CEA on both the surgical side (*R* = 0.56, *P* < 0.001), and the nonsurgical side (*R* = 0.51, *P* < 0.001) within the 5-mm circles. Therefore, we believe that the change in CC thickness is reflective of reperfusion of the choroid after CEA and may be an even more direct measurement of choroidal perfusion than CT measurements that include changes in both the choroidal vessels and stroma.

Interestingly, from the two exemplary cases we showed above, the thickness maps of the CC and choroid in our series suggest that the region with the greatest increase in CC thickness did not necessarily align with the area of increased CT. Because the largest vessels in the choroid are the choroidal veins and the CT correlated with the choroidal vascular volume,^12^ this difference in thickness changes between the CC and choroid may have to do with the drainage pattern of the CC into the choroidal veins; however, the exact mechanism for this difference requires additional investigation.

We also observed another intriguing phenomenon in the two illustrative patients whose eyes on the contralateral side of CEA (Figs. 3, 4) had a slight reduction in CC FD% and an increase in CC thickness and CT after the procedure (more pronounced in the second case); however, this occurrence was not statistically significant across all cases in our series. There is no direct evidence in the literature regarding the change of CC perfusion on the contralateral side after CEA, several previous studies investigating CT changes on both sides after unilateral CEA suggested a simultaneous improvement of choroidal perfusion on the contralateral side and this improvement was typically less pronounced than that on the ipsilateral side.^17^^,^^18^ This bilateral improvement was thought to be due to increased cerebral blood flow with redistribution of flow through the circle of Willis and the ophthalmic arteries.^51^ In addition, Wang et al.^52^ demonstrated bilateral improvement of ocular blood flow using Doppler ultrasound, and the primary collateral pathway contributing to the improved perfusion on the contralateral side in their study was believed to be cross-flow via the anterior communicating artery.^53^ However, the existence of this phenomenon is still up for debate, as some other studies have failed to observe any improvement in CT on the contralateral side. Therefore, the exact mechanism underlying the improvement of CC perfusion on the nonsurgical side in some individual cases requires further investigation.

We also examined the effect of various potential confounders, including age, MAP, extent of stenosis, and the presence of comorbidities such as diabetes, hypertension, and smoking status, on CC perfusion. We did not find age, MAP, or the presence of diabetes or hypertension to be associated with the changes in CC FD% and CC thickness measurements after CEA on either the surgical side or the nonsurgical side. However, we found that smoking status had a significantly positive impact on CC FD% changes and a significant negative impact on CC thickness change on both the surgical side and the nonsurgical side before and after CEA. This suggests that smokers experienced less improvement in CC perfusion, possibly attributed to factors such as increased arterial wall stiffness and peripheral vasoconstrictive effects.^17^ Additionally, the extent of stenosis had a positive correlation with the changes in CC FD% in our series, indicating that the patients with more severe stenosis experienced less CC perfusion improvement after CEA. However, the CC thickness change after CEA was not influenced by the degree of stenosis. Hence, it was still inconclusive how the degree of stenosis affects reperfusion of CC perfusion. Akca Bayar et al.^16^ evaluated the CT change after carotid artery surgery by subdividing their patients into two groups according to the extent of their stenosis. They found a significant increase in CT in patients with 50% to 70% stenosis, but not in patients with >70% stenosis at any time after carotid surgeries. This may suggest an irreversibility in choroidal thinning from longstanding severe CAS or downstream stenosis in the OA that may prevent reperfusion. However, a study by Vanninen et al.^54^ that examined the hemodynamic effect of CEA on flow rates in the carotid artery using magnetic resonance flow quantification found that there appeared to be greater improvement in the flow rate on the surgical side with the severe stenosis at the carotid artery bifurcation compared with mild to moderate stenosis at the carotid artery bifurcation. This evidence suggests that the greatest response from CEA should be expected following treatment for more severe CAS. One limitation of our study is the relatively homogeneous degree of stenosis among our patients. The majority of our patients had more than 70% stenosis (severe stenosis), with only three out of 56 patients having less than 70% stenosis on the surgical side Therefore, it is challenging to definitely address the ongoing debate regarding the influence of stenosis severity on CC perfusion. One other possibility for the absence of CC changes after the procedure in some cases may be flow impairment to the eye downstream from where the CAS is located, possibly within the OA.^55^^,^^56^

Our study has several strengths, including the use of SS-OCTA imaging for visualization of the CC and the use of validated semi-automated algorithms to analyze CC FD% and CC thickness.^27^^,^^29^^,^^34^ Another major strength of our study is that, to the best of our knowledge, it is the first to explore changes in CC perfusion following CEA, shedding light on the impact of this vascular intervention on ocular microvascular perfusion. This is particularly important because CC perfusion is critical for the health of the RPE and photoreceptors and has been implicated as playing a role in different diseases, particularly AMD.^28^^,^^57^ Hibert et al.^55^ reported significantly decreased volumetric OA flow rates and evidence of stenosis at the ostium of the OA in patients with late AMD, which may explain the lack of impact from CEA on choroidal and CC measurements in some patients. Furthermore, Lylyk et al.^56^ reported on the subjective benefit of OA cannulation in patients with late AMD. Our research offers additional evidence for the potential impact of upstream vascular therapy in modifying CC perfusion. This discovery may pave the way for alternative treatments for retinal diseases that exhibit diminished CC perfusion.

There are also several limitations to our study. First, the study was conducted over a relatively short period of time with an average follow-up of only 3 days. Therefore, it is unclear whether the observed improvements in ocular perfusion, particularly the CC perfusion change, can be sustained over a longer period. Longer follow-up of this cohort would be necessary to establish the potential long-term benefits of CEA in improving ocular perfusion. Second, when introducing novel biomarkers to assess the impact of CEA on ocular blood flow, it is advisable to compare them with existing methods such as OA Doppler ultrasound. However, it is important to mention that evaluating OA flow immediately after CEA is not a routine practice for vascular surgery, primarily due to its technical limitation and availability. Nonetheless, it holds promise as a subject for future exploration to prove that the OCT biomarkers are consistent with OA Doppler ultrasound outcomes and potentially could be used in lieu of ultrasound measurements. In addition, ocular flow rate is an important parameter in evaluating ocular perfusion after CEA and can be measured by color Doppler or MRI. It would be intriguing to conduct velocimetry measurements on the CC as another biomarker for studying CC perfusion. This could be accomplished by OCTA using the VISTA algorithm^58^ or capillary velocimetry strategy.^59^ Future studies should also investigate the impact of carotid revascularization on functional outcomes such as changes in normal and low-luminance visual acuity, contrast sensitivity, reading speed, dark adaptometry rod intercept times, and microperimetry thresholds. Moreover, it would be worth exploring the changes of CC perfusion in subjects with and without AMD after CEA and the association of these changes with functional outcomes.

In summary, a significant reduction in mean CC FD% and an increase in CC thickness measurements were observed in eyes on the ipsilateral side of clinically significant CAS following CEA, suggesting an improvement in CC perfusion after carotid artery revascularization. Studying changes in CC perfusion can advance our understanding of how carotid artery revascularization directly affects microvascular perfusion in the eye and if these OCT measurements of CC perfusion can serve as novel biomarkers for evaluating the extent of ocular ischemia and visual function. Moreover, exploring this line of research may uncover valuable information regarding the effectiveness of upstream vascular interventions, such as carotid artery or ophthalmic artery recanalization, as options for treating retinal disorders characterized by compromised CC function.

## References

[1] Flaherty ML, Kissela B, Khoury JC, et al. Carotid artery stenosis as a cause of stroke. *Neuroepidemiology*. 2013; 40: 36–41. 23075828 10.1159/000341410 PMC3626492

[2] Lamanna A, Maingard J, Barras CD, et al. Carotid artery stenting: current state of evidence and future directions. *Acta Neurol Scand*. 2019; 139: 318–333. 30613950 10.1111/ane.13062

[3] Bonati LH, Jansen O, de Borst GJ, Brown MM. Management of atherosclerotic extracranial carotid artery stenosis. *Lancet Neurol*. 2022; 21: 273–283. 35182512 10.1016/S1474-4422(21)00359-8

[4] Nana P, Spanos K, Antoniou G, et al. The effect of carotid revascularization on the ophthalmic artery flow: systematic review and meta-analysis. *Int Angiol*. 2021; 40: 23–28. 32892613 10.23736/S0392-9590.20.04448-X

[5] Sekine T, Takagi R, Amano Y, et al. 4D flow MR imaging of ophthalmic artery flow in patients with internal carotid artery stenosis. *Magn Reson Med Sci*. 2018; 17: 13–20. 28367905 10.2463/mrms.mp.2016-0074 PMC5760228

[6] Kawaguchi S, Iida J, Uchiyama Y. Ocular circulation and chronic ocular ischemic syndrome before and after carotid artery revascularization surgery. *J Ophthalmol*. 2012; 2012: 350475. 23316337 10.1155/2012/350475 PMC3536051

[7] Mendrinos E, Machinis TG, Pournaras CJ. Ocular ischemic syndrome. *Surv Ophthalmol*. 2010; 55: 2–34. 19833366 10.1016/j.survophthal.2009.02.024

[8] Murad MH, Shahrour A, Shah ND, Montori VM, Ricotta JJ. A systematic review and meta-analysis of randomized trials of carotid endarterectomy vs stenting. *J Vasc Surg*. 2011; 53: 792–797. 21216556 10.1016/j.jvs.2010.10.101

[9] Fujioka S. Use of orbital color Doppler imaging for detecting internal carotid artery stenosis in patients with amaurosis fugax. *Jpn J Ophthalmol*. 2003; 47: 276–280. 12782164 10.1016/s0021-5155(03)00016-9

[10] Emiroglu MY, Evlice M, Akcakoyun M, et al. Effects of obstructive carotid artery disease on ocular circulation and the safety of carotid artery stenting. *Heart Lung Circ*. 2017; 26: 1069–1078. 28162948 10.1016/j.hlc.2016.11.020

[11] Michalinos A, Zogana S, Kotsiomitis E, Mazarakis A, Troupis T. Anatomy of the ophthalmic artery: a review concerning its modern surgical and clinical applications. *Anat Res Int*. 2015; 2015: 591961. 26635976 10.1155/2015/591961 PMC4655262

[12] Zhou H, Dai Y, Shi Y, et al. Age-related changes in choroidal thickness and the volume of vessels and stroma using swept-source OCT and fully automated algorithms. *Ophthalmol Retina*. 2020; 4: 204–215. 32033714 10.1016/j.oret.2019.09.012 PMC7812781

[13] Invernizzi A, Pellegrini M, Cornish E, Yi Chong Teo K, Cereda M, Chabblani J. Imaging the choroid: from indocyanine green angiography to optical coherence tomography angiography. *Asia Pac J Ophthalmol (Phila)*. 2020; 9: 335–348. 32739938 10.1097/APO.0000000000000307

[14] Wan J, Kwapong WR, Tao W, et al. Choroidal changes in carotid stenosis patients after stenting detected by swept-source optical coherence tomography angiography. *Curr Neurovasc Res*. 2022; 19: 100–107. 35388758 10.2174/1567202619666220406092532

[15] Biberoglu E, Eraslan M, Midi I, Baltacioglu F, Bitargil M. Ocular blood flow and choroidal thickness changes after carotid artery stenting. *Arq Bras Oftalmol*. 2020; 83: 417–423. 33084820 10.5935/0004-2749.20200081 PMC12289278

[16] Akca Bayar S, Kayaarasi Ozturker Z, Pinarci EY, Ercan ZE, Akay HT, Yilmaz G. Structural analysis of the retina and choroid before and after carotid artery surgery. *Curr Eye Res*. 2020; 45: 496–503. 31507205 10.1080/02713683.2019.1666994

[17] Krytkowska E, Masiuk M, Kawa MP, et al. Impact of carotid endarterectomy on choroidal thickness and volume in enhanced depth optical coherence tomography imaging. *J Ophthalmol*. 2020; 2020: 8326207. 32280535 10.1155/2020/8326207 PMC7125458

[18] Lareyre F, Nguyen E, Raffort J, et al. Changes in ocular subfoveal choroidal thickness after carotid endarterectomy using enhanced depth imaging optical coherence tomography: a pilot study. *Angiology*. 2018; 69: 574–581. 29082746 10.1177/0003319717737223

[19] Ala-Kauhaluoma M, Koskinen SM, Silvennoinen H, et al. Subfoveal choroidal thickness in ipsi- and contralateral eyes of patients with carotid stenosis before and after carotid endarterectomy: a prospective study. *Acta Ophthalmol*. 2021; 99: 545–552. 33354923 10.1111/aos.14648

[20] Rabina G, Barequet D, Mimouni M, et al. Carotid artery endarterectomy effect on choroidal thickness: one-year follow-up. *J Ophthalmol*. 2018; 2018: 8324093. 30662767 10.1155/2018/8324093 PMC6312583

[21] Zhang Y, Zhou SW, Noam N, et al. Influence of carotid endarterectomy on choroidal perfusion: the INFLATE Study [published online ahead of print July 31, 2023]. *Ophthalmol Retina*, 10.1016/j.oret.2023.07.026. 37531996

[22] Lejoyeux R, Benillouche J, Ong J, et al. Choriocapillaris: fundamentals and advancements. *Prog Retin Eye Res*. 2022; 87: 100997. 34293477 10.1016/j.preteyeres.2021.100997

[23] Mulfaul K, Russell JF, Voigt AP, Stone EM, Tucker BA, Mullins RF. The essential role of the choriocapillaris in vision: novel insights from imaging and molecular biology. *Annu Rev Vis Sci*. 2022; 8: 33–52. 36108103 10.1146/annurev-vision-100820-085958 PMC9668353

[24] Hayreh SS. The choriocapillaris. *Albrecht Von Graefes Arch Klin Exp Ophthalmol*. 1974; 192: 165–179. 4219556 10.1007/BF00416864

[25] Nickla DL, Wallman J. The multifunctional choroid. *Prog Retin Eye Res*. 2010; 29: 144–168. 20044062 10.1016/j.preteyeres.2009.12.002 PMC2913695

[26] Zhang Q, Zheng F, Motulsky EH, et al. A novel strategy for quantifying choriocapillaris flow voids using swept-source OCT angiography. *Invest Ophthalmol Vis Sci*. 2018; 59: 203–211. 29340648 10.1167/iovs.17-22953 PMC5770182

[27] Shi Y, Chu Z, Wang L, et al. Validation of a compensation strategy used to detect choriocapillaris flow deficits under drusen with swept source OCT angiography. *Am J Ophthalmol*. 2020; 220: 115–127. 32621895 10.1016/j.ajo.2020.06.033 PMC8063776

[28] Shi Y, Zhang Q, Zheng F, et al. Correlations between different choriocapillaris flow deficit parameters in normal eyes using swept source OCT angiography. *Am J Ophthalmol*. 2020; 209: 18–26. 31562858 10.1016/j.ajo.2019.09.017 PMC7017580

[29] Zhou H, Dai Y, Gregori G, et al. Automated morphometric measurement of the retinal pigment epithelium complex and choriocapillaris using swept source OCT. *Biomed Opt Express*. 2020; 11: 1834–1850. 32341851 10.1364/BOE.385113 PMC7173887

[30] Naylor AR, Rothwell PM, Bell PR. Overview of the principal results and secondary analyses from the European and North American randomised trials of endarterectomy for symptomatic carotid stenosis. *Eur J Vasc Endovasc Surg*. 2003; 26: 115–129. 12917824 10.1053/ejvs.2002.1946

[31] Zhang Q, Zhang A, Lee CS, et al. Projection artifact removal improves visualization and quantitation of macular neovascularization imaged by optical coherence tomography angiography. *Ophthalmol Retina*. 2017; 1(2): 124–136. 28584883 10.1016/j.oret.2016.08.005 PMC5455345

[32] Chu Z, Chen CL, Zhang Q, et al. Complex signal-based optical coherence tomography angiography enables in vivo visualization of choriocapillaris in human choroid. *J Biomed Opt*. 2017; 22: 1–10. 10.1117/1.JBO.22.12.121705 PMC5745879 29178697

[33] Chu Z, Zhang Q, Zhou H, et al. Quantifying choriocapillaris flow deficits using global and localized thresholding methods: a correlation study. *Quant Imaging Med Surg*. 2018; 8: 1102–1112. 30701164 10.21037/qims.2018.12.09 PMC6328379

[34] Zhang Q, Shi Y, Zhou H, et al. Accurate estimation of choriocapillaris flow deficits beyond normal intercapillary spacing with swept source OCT angiography. *Quant Imaging Med Surg*. 2018; 8: 658–666. 30211033 10.21037/qims.2018.08.10 PMC6127524

[35] Chu Z, Gregori G, Rosenfeld PJ, Wang RK. Quantification of choriocapillaris with optical coherence tomography angiography: a comparison study. *Am J Ophthalmol*. 2019; 208: 111–123. 31323202 10.1016/j.ajo.2019.07.003 PMC6889046

[36] Chu Z, Zhang Q, Gregori G, Rosenfeld PJ, Wang RK. Guidelines for imaging the choriocapillaris using OCT angiography. *Am J Ophthalmol*. 2021; 222: 92–101 32891694 10.1016/j.ajo.2020.08.045 PMC7930158

[37] Zhou H, Chu Z, Zhang Q, et al. Attenuation correction assisted automatic segmentation for assessing choroidal thickness and vasculature with swept-source OCT. *Biomed Opt Express*. 2018; 9: 6067–6080. 31065413 10.1364/BOE.9.006067 PMC6490991

[38] Lu CD, Lee B, Schottenhamml J, Maier A, Pugh EN Jr, Fujimoto JG. Photoreceptor layer thickness changes during dark adaptation observed with ultrahigh-resolution optical coherence tomography. *Invest Ophthalmol Vis Sci*. 2017; 58: 4632–4643. 28898357 10.1167/iovs.17-22171 PMC5596796

[39] Zhou H, Lu J, Chen K, et al. Mitigating the effects of choroidal hyper- and hypo-transmission defects on choroidal vascularity index assessments using optical coherence tomography. *Quant Imaging Med Surg*. 2022; 12: 2932–2946. 35502369 10.21037/qims-21-1093 PMC9014140

[40] R Core Team. *R: A Language and Environment for Statistical Computing*. Vienna, Austria: R Foundation for Statistical Computing; 2022.

[41] Rosner B, Glynn RJ, Lee ML. Extension of the rank sum test for clustered data: two-group comparisons with group membership defined at the subunit level. *Biometrics*. 2006; 62: 1251–1259. 17156300 10.1111/j.1541-0420.2006.00582.x

[42] Jiang Y, Lee MT, He X, Rosner B, Yan J. Wilcoxon rank-based tests for clustered data with R package clusrank. *J Stat Softw*. 2020; 96(6): 1–26.

[43] Pierro L, Arrigo A, De Crescenzo M, et al. Quantitative optical coherence tomography angiography detects retinal perfusion changes in carotid artery stenosis. *Front Neurosci*. 2021; 15: 640666. 33967678 10.3389/fnins.2021.640666 PMC8100533

[44] Zheng F, Zhang Q, Shi Y, et al. Age-dependent changes in the macular choriocapillaris of normal eyes imaged with swept-source optical coherence tomography angiography. *Am J Ophthalmol*. 2019; 200: 110–122. 30639367 10.1016/j.ajo.2018.12.025 PMC6513331

[45] Olver JM. Functional anatomy of the choroidal circulation: methyl methacrylate casting of human choroid. *Eye (Lond)*. 1990; 4(pt 2): 262–272. 2379644 10.1038/eye.1990.38

[46] Choi W, Mohler KJ, Potsaid B, et al. Choriocapillaris and choroidal microvasculature imaging with ultrahigh speed OCT angiography. *PLoS One*. 2013; 8: e81499. 24349078 10.1371/journal.pone.0081499 PMC3859478

[47] Leskova W, Watts MN, Carter PR, Eshaq RS, Harris NR. Measurement of retinal blood flow rate in diabetic rats: disparity between techniques due to redistribution of flow. *Invest Ophthalmol Vis Sci*. 2013; 54: 2992–2999. 23572104 10.1167/iovs.13-11915 PMC3638664

[48] Arya M, Filho MB, Rebhun CB, et al. Analyzing relative flow speeds in diabetic retinopathy using variable interscan time analysis OCT angiography. *Ophthalmol Retina*. 2021; 5: 49–59. 32585373 10.1016/j.oret.2020.06.024 PMC8906440

[49] Koch R, Seto B, Yamada K, et al. Relative retinal blood flow: a novel and informative measure of unilateral retinal vein occlusion severity. *Transl Vis Sci Technol*. 2021; 10: 15. 10.1167/tvst.10.3.15 PMC7961123 34003949

[50] Pinhas A, Migacz JV, Zhou DB, et al. Insights into sickle cell disease through the retinal microvasculature: adaptive optics scanning light ophthalmoscopy correlates of clinical OCT angiography. *Ophthalmol Sci*. 2022; 2: 100196. 36531581 10.1016/j.xops.2022.100196 PMC9754983

[51] Vriens EM, Wieneke GH, Hillen B, Eikelboom BC, Van Huffelen AC, Visser GH. Flow redistribution in the major cerebral arteries after carotid endarterectomy: a study with transcranial Doppler scan. *J Vasc Surg*. 2001; 33: 139–147. 11137934 10.1067/mva.2001.109768

[52] Wang J, Wang W, Jin B, et al. Improvement in cerebral and ocular hemodynamics early after carotid endarterectomy in patients of severe carotid artery stenosis with or without contralateral carotid occlusion. *Biomed Res Int*. 2016; 2016: 2901028. 27642593 10.1155/2016/2901028 PMC5011514

[53] Baracchini C, Meneghetti G, Manara R, Ermani M, Ballotta E. Cerebral hemodynamics after contralateral carotid endarterectomy in patients with symptomatic and asymptomatic carotid occlusion: a 10-year follow-up. *J Cereb Blood Flow Metab*. 2006; 26: 899–905. 16395290 10.1038/sj.jcbfm.9600260

[54] Vanninen R, Koivisto K, Tulla H, Manninen H, Partanen K. Hemodynamic effects of carotid endarterectomy by magnetic resonance flow quantification. *Stroke*. 1995; 26: 84–89. 7839404 10.1161/01.str.26.1.84

[55] Hibert ML, Chen YI, Ohringer N, et al. Altered blood flow in the ophthalmic and internal carotid arteries in patients with age-related macular degeneration measured using noncontrast MR angiography at 7T. *AJNR Am J Neuroradiol*. 2021; 42: 1653–1660. 34210664 10.3174/ajnr.A7187 PMC8423057

[56] Lylyk I, Bleise C, Lylyk PN, et al. Ophthalmic artery angioplasty for age-related macular degeneration. *J Neurointerv Surg*. 2022; 14: 968–972. 34987072 10.1136/neurintsurg-2021-018222 PMC9484375

[57] Rosenfeld PJ, Trivizki O, Gregori G, Wang RK. An update on the hemodynamic model of age-related macular degeneration. *Am J Ophthalmol*. 2022; 235: 291–299. 34509436 10.1016/j.ajo.2021.08.015

[58] Arya M, Rashad R, Sorour O, Moult EM, Fujimoto JG, Waheed NK. Optical coherence tomography angiography (OCTA) flow speed mapping technology for retinal diseases. *Expert Rev Med Devices*. 2018; 15: 875–882. 30460869 10.1080/17434440.2018.1548932 PMC6529293

[59] Wang RK, Zhang Q, Li Y, Song S. Optical coherence tomography angiography-based capillary velocimetry. *J Biomed Opt*. 2017; 22: 066008 28617921 10.1117/1.JBO.22.6.066008 PMC5472241

